# Letter from Dr. Beaugrand

**Published:** 1839-08-31

**Authors:** 


### FOREIGN CORRESPONDENCE.

### LETTER FROM DR. BEAUGRAND.—No. III.

### *M. Dubois's Lecture on the Theory of Infection and Contagion.*

Paris, *4th July*, 1839.

To the Editors of the Medical Examiner.

The governments of Europe are still so much behind the spirit of the age in legislation upon sanitary subjects, that we cannot but hail with satisfaction every effort to illuminate so important a question. We shall, we think, be rendering a real service to science, by publishing the lecture which M. Dubois, of Amiens, adjunct professor in the Faculty of Medicine, of Paris, has been lately delivering upon the theory of infection and contagion.

While treating in general of the etiology of the subject, M. Dubois, of course, entered fully into the important question of its *specific causes.* This question he handled, we feel bound to say, with a lucidity and independence, honourable alike to his talents and his character. The *specific causes* he defines to be those which produce, in individuals exposed to their action, a well ascertained train of morbid phenomena—a disease, the principal features of which, with the order of their development, may be determined beforehand. Hence it follows, that the features from which the specific character is derived, are to be found not in the causes themselves, but in their effects. That is, the closest relations exist between the nature of these causes and the pathological phenomena which result from them. This speciality of action is such, that, from a given cause, an enlightened observer is able to prognosticate the *probable* effects. We say probable, for, from idiosyncracies, there are subjects unsusceptible to the action of specific agents.

Specific causes are of three kinds. Some do not pass the limits of mere intoxication; others produce actual infection; and others, again, give rise to contagion. In this classification, it is perceived, there is a sort of gradation. In the first case, the cause poisons the individual, but *goes no further.* Whatever be the severity of the effects produced, whatever the intensity of the symptoms, the sphere of activity remains confined to the particular subject affected. It must be observed, that, in this case, the cause is always material, and appreciable by the senses, and the ordinary means of analysis.

In the second case, the cause poisons both *the individual and his locality;* so that other healthy individuals, brought within it, may contract the same disease. Here, the sphere of action is enlarged; it constitutes what is termed a *focus of infection*, of which the real or supposed active principle escapes investigation.

In the third case, so great is the intensity of the cause, or, to speak more correctly, such is its power of reproduction, that it reappears in all the bodies which it affects, so as to transmit the disease without a focus of infection.

We shall not follow the professor in his examination of the first class of specific agents, which are, from their definition, neither more nor less than poisons. This subject is familiar to every one, and amply discussed in treatises on toxicology. We pass to the second order of specific causes.

*Of Infection.*—We have said that the agent producing intoxication is material: that of infection is not tangible. This principle is generally designated by the term *miasm*,—that of contagion, we shall presently see, is a *virus.* From the confounding of these terms, (miasm and virus,) infection and contagion have not been accurately distinguished from each other. Miasm, the agent of infection, is the result of circumstances belonging to localities; it may likewise proceed from living and diseased bodies; but it always retains such a local character, as to constitute a centre of radiation. It is a matter of observation, that miasmata can act upon individuals only after having formed a focus of infection. It must here be remarked, that infectious principles are not all to be traced to the same origin,—they must be subdivided. Some, in fact, emanate from certain localities, as marshes, collections of dirt, stagnant waters, masses of organized substances, animal or vegetable, in a state of putrefaction. Others emanate from living bodies, crowded together in narrow limits, as hospitals, prisons, camps, besieged cities, &c. The foci thus formed have, however, appreciable limits, beyond which the morbific principle is extinguished and disappears. It may be readily inferred from what has been said, that miasmata, dependent on the circumstances of localities, can form foci only in spots where these circumstances exist permanently or periodically. Thus, the miasmata which emanate from marshes, the effect of which is to produce paroxysmal fevers, can constitute foci only in the neighbourhood of these same marshes. The miasmata which are developed in the low lands of the Antilles, Lower Mexico, Vera Cruz, New Orleans, Charleston,

&c., and which develope fevers of a much more serious character, have likewise the sphere of their action limited to the places which give them birth.

A very remarkable fact, not generally understood in Europe by physicians, is, that, whatever be the number of individuals ill from the effects of these miasmata, if they are transported beyond the limits of the focus, they do not carry with them the sources of the morbific principle, and they cannot elsewhere form the nucleus of a fresh focus of infection. If, under particular circumstances, and at occasional periods, diseases have been known to prevail in regions heretofore beyond the limits of the noxious centres, it is because temporarily local dispositions, of a parallel nature, have been established, have produced miasmata in these localities, and accidentally formed there foci of infection.

But it is not the same with miasmata occasioned by the crowding together of diseased bodies. These may *every where* form centres of morbific radiation, whatever be the general condition of the localities. It is in this case essential that they should form a focus; otherwise, they are altogether harmless; a solitary individual cannot carry with him a sufficiently large dose of the noxious principle, to communicate the disease. We have examples of this in the typhus of armies, puerperal fevers, &c. But, let the patients be transported in mass, and it is quite otherwise: they do not cease to be surrounded by the same disastrous circumstances which originated the disease with which they are affected,—they form a morbific train, scattering disease through the regions which they traverse. Such was the case in France, in 1814, from Mayence to Paris, when the army, forced to keep up a retreating fight, successively filled and evacuated the different hospitals, and left typhus fever every where upon their route.

The infectious principle, once admitted into the economy, is an actual poisoning,—but it is a miasmatic one, not produced by any material agent, solid, liquid, or gaseous. The effects produced here are of a different character, though in some cases analogous with the effects of septic poisons, that is to say, those that produce alterations in the liquids of the economy.

But, since miasmata are not appreciable by our senses and methods of analysis, does it follow that they have no actual existence? By no means. In the first place, we know their source and origin; we know the limits which they occupy in certain localities; we know that they may be absorbed, and that a certain time elapses between the period of their absorption, and the appearance of the symptoms which denote it. For the solution, then, of this interesting problem, we have three points given:—1st, the circumstances which give rise to the development of miasmata; 2dly, their topography and geography; 3dly, the mode of their introduction into the economy, and the effects which result therefrom.

Taking up, successively, each of these points, M. Dubois shows that the origin of miasmata is always to be traced to organized matter, particularly when vegetable or animal matter is in a state of decomposition. Hence, the danger from marshes, common-sewers, ponds, and from certain low situations, &c. &c. Finally, he enters into the important pathological question, relative to the period during which the miasm remains in the economy, before manifesting its effects; in a word, the duration of the *incubation*. This must necessarily vary, both from the character of the miasm, and the constitution of the individual. Other things being the same, the period of incubation is shorter when the miasmata emanate from the congregation of diseased bodies. The same result is observed when organic matter in a state of putrefaction, producing the miasmata, is exposed to the influence of a burning sun. Their activity is carried to the highest degree when they are produced at the same time by local conditions, and the collection of bodies. We must here again dwell upon the fact, that, in every case, for the disease to be developed, an individual must be exposed to the miasmata, within the limits of a focus of infection—this is an indispensable condition. One circumstance has led many physicians into error, namely, that the disease may be developed and run through its stages, *without* the limits of the focus of infection; but, in all these cases, the patient must have changed his locality during the period of incubation. He absorbs the miasm in an infected spot, and carries with him the germ of the disease, which sooner or later attacks him. We shall terminate our remarks upon the subject of infection, by observing, that the first effects of the infectious principle show themselves upon the condition of the liquids of the economy and of the nervous system. This alteration of the liquids may go so far as to change their physical aspect. Sometimes the nervous system is the first affected; we then have debility, and extreme prostration of the powers of the mind and body. Generally these two orders of phenomena exist simultaneously.

*Of Contagion.* The opinions of authors are far from being united upon this subject. Hufeland maintained that every specific disease, determined by a subtile matter introduced into the economy, was a contagious disease. Thus, with him, there would be no difference between a poisoning produced by hydrocyanic acid, for example, and a contagious disease of the highest grade. Other pathologists, Dr. Chomel among the number, hold that the properties of contagious principles are, to determine in the individuals attacked, the reproduction of principles similar to themselves, and capable of producing the same effects in another person. This definition is not accurate, and we know that the miasmata produced by collections of the sick, are capable of exciting in other persons both the same morbid phenomena, and principles similar to themselves, and yet there is no contagion. Hence, the error of many physicians in placing the typhus of camps in the list of contagious affections. If, now, we examine the ancient theory of Fracastor, we shall see that it is absurd in many points, and requires modification in others. According to this author, in the diseases of which we speak, all causes act in the same manner. There is a virus, eminently subtile, escaping from the sick bodies, attaching itself by preference to vegetable or animal substances, such as wool, cloths, straw, gliding into letters, insinuating itself into cotton and silk stuffs, and susceptible of preservation for thirty or forty years, of crossing the seas, and of being transported to great distances. Upon this theory depend all the sanitary measures at present in force. Every thing, according to Fracastor, is contagious; he allows no cases of infection. Other doctrines have been put forth upon this subject, but we have not space here to speak of them.

With M. Dubois, the principle of contagion is a *virus*, and he lays down several kinds of them. Some are called *fixed*, when they appear to enter into the constitution of the animal tissues; such is the syphilitic virus. Others are susceptible of being disseminated in the air, circulating like miasmata. But, what it is particularly important to establish, are the different modes by which this transmission may be effected. It will be seen that there is, in this particular, a notable difference. Sometimes the virus is so fixed, that contagion can take place only after an actual inoculation; such is the virus of rabies. At other times, actual propinquity is indeed necessary, but simple contact upon an absorbing surface, a mucous membrane, for example, is sufficient; the syphilitic virus acts in this manner. In other cases, simple contact upon a cutaneous surface produces the effect; this is the condition necessary for the action of the psoric virus. This last example seems to us not to have been happily selected by the professor, for many physicians, ourselves among the number, attribute the contagiousness of the itch to the *acarus;*—we shall see below that M. Dubois discusses this question. At other times, the transmission of the virus takes place at a distance, by the medium of the atmosphere, as in the eruptive fevers, variola, scarlatina, &c. Sometimes, it is effected by means of contaminated objects, *contactu rei contaminatæ*, as Van Helmont observes with reference to the plague, which, it is said, may be communicated in this manner. In fine, the same disease may be transmissible by several of the modes which we have enumerated—the plague, for instance, according to the testimony of various observers. It may be propagated by means of inoculation, by immediate and mediate contact, and, as above remarked, by contaminated objects. The difference in the mode of transmission has no connection with the gravity or duration of the disease.

Here arises a question, unsusceptible of solution in the actual state of our knowledge—What is a virus? Does the material object which we see owe its virulent properties to a principle of which it is merely the vehicle? Has the virus a separate, distinct existence? Or does the material substance owe its property of transmission merely to the chemical combination of its constituent elements? Or does its virulence, in every case, depend on the presence of imperceptible animalcules? M. Dubois has attempted some experiments to determine the composition of a particular virus—the vaccine. The following are the principal results of his experiments:—If, immediately after extracting, at the proper time, the vaccine virus from pustules, normally developed, a drop be subjected to microscopic examination, it is found to be perfectly limpid and transparent. There is not the least appearance of globules, granulations, or scales. Soon there are seen to form, very elegant crystals of muriate of ammonia; finally, there is established a peculiar mode of desiccation. There are seen lines of fibres (*fibrilles*) folded upon each other, very different from the crevices observed in every drop of dried albumen. M. Dubois has, besides, arrived at other more important results. The vaccine virus, congealed, (he has reduced it to a temperature of 10° below zero of Réaumur,) loses its property of transmission. The same result takes place if it be raised to the temperature of boiling water. In these cases, how do the two extremes of temperature act? Is it by killing the imperceptible animalcules, or simply by altering the constitution of the virus? These are questions which cannot be solved, whatever conclusions we may reach respecting the difference of effects produced by each distinct virus.

Let us now pass to some considerations upon the different characters belonging to the principles of contagion and infection. Every disease, produced by a contagious principle, is itself contagious. We know that such is not the case with affections depending on miasmata, and that the transportation of sick persons beyond the limits of a *focus of infection*, has no power to carry with it the germ of the disease. On the contrary, every individual affected with a contagious disease, becomes capable of spreading it, of communicating it to others, whatever may be the salubrity of the places to which he may go. Hence we deduce the important conclusion, that, without the intervention of a focus of contagion, a disease may be transmitted from diseased to healthy individuals, and, further, from a single contaminated individual, to an entire population. This is the important, distinctive character of contagion, which, as it is better understood, and better studied, is every day narrowing the circle of contagious affections. Grave epidemics, serious public calamities, gave occasion to the adoption of the doctrines of Fracastor; and the sanitary regulations which resulted from them, were indiscriminately applied to all the contagious diseases—to those of which the principle is fixed, as well as to those in which it is susceptible of dissemination; and, finally, to simple infections, dependent on localities. But, as physicians have become more enlightened, they have been impressed with the necessity of proper distinctions, that, while acknowledging the existence of affections really contagious, it would be absurd to apply to those of which the virus is fixed,—syphilis, for example,—regulations appropriated to the plague. In later times, they have seen that the focus of the infection is confined to certain limits, which it does not pass, even with the emigration of the sick; distinguishing always between infections dependent on localities, and not going beyond their limits, and those resulting from collections of the sick, which may be reproduced at any time and place. We have dwelt particularly upon these points, and have insisted upon them more than once, under the conviction that they are of the highest importance, and are destined to effect a revolution in the sanitary systems generally adopted. We indulge the hope that diseases, which are spread by a contagious virus, will no longer be confounded with those maintained by a *local, circumscribed* disposition, and that the unfortunate inhabitants of those localities will cease to be shut up and condemned to perish, as in a tomb.

Here a question offers itself for examination—Is there no other distinction between the principles of contagion and of infection than that which results from the degree of intensity, or are there distinctions depending upon the very nature of those principles? Several circumstances tend to the belief that there are other distinctions than those which depend upon the degree of activity. In the first place, there are contagious diseases, much less grave than certain infectious diseases. Among the first are, some of the eruptive fevers; among the latter, the camp-typhus. There is, therefore, something characteristic, something special, in certain contagious or infectious principles, which distinguishes them from others of their class, and gives them a character of severity or mildness not belonging to the latter.

We have thrown out the idea that, in miasmata and virus, there was probably a sort of animation; however, the active existence of animated beings has only been established in particular virus. Analogy would lead to the belief, as has been remarked by M. Raspail, that these beings might exist in other virus, fixed or circulatory. It is well known, that, since the discovery of the acarus scabiei, some pathologists have persisted in viewing this as merely a complication of the affection. According to M. Raspail, however, it can be no longer a matter of dispute that this animal is the actual cause of the symptoms. We must not, however, confound the infusoria which exist as consecutive products in animal fluids, with the animalcula which propagate contagious diseases. M. Donné appears to have fallen into this error. M. Donné having discovered animalcula in the vaginal fluid, attempted thence to deduce a distinction between contagious and non-contagious discharges. M. Raspail, however, at once recognised here the simple presence of an infusorium, already observed by Müller in the maceration of flesh. Thus, a mere parasite had been mistaken for the source of disease, which was imputed to the animalcula, the harmless fruits of a preceding putrefaction.

As to miasmata, it has been already said that they arise from organized matter, in a state either of life or decomposition. May it not hence be inferred that they contain something organized? In the second place, they attack directly the principle of life, and, finally, they require, before acting, a certain period of incubation. Have we not here the proof of a kind of animation which requires time to take up relations with the organization into which the miasm has penetrated?

In the cure both of contagious and infectious principles, regard must be had both to individual idiosyncracies and to certain external circumstances. The influence of the first is indisputable. It is well known that certain individuals are so fortunate as to be unsusceptible of the action of virus, at least of many of them. The condition termed acclimation, is also known to be a protection against the influence of miasmata. Such are also said to be the effects of serenity of temper, sobriety, moderate exercise, &c. With regard to external circumstances, we are told that much depends upon the condition of the atmosphere,—that an elevated temperature favours the action of a virus circulating in the air. But this is a question for further examination, inasmuch as the assertion has been based upon a want of distinction between miasmata and virus.

Without here entering again upon the arguments confirmatory of the views of M. Raspail, touching the existence of animalcula, at least in some virus, we shall add, that the remarkable property belonging to certain contagious principles, of being propagated and preserved by means of inoculation, is a fresh point of resemblance to beings having a distinct existence; for, after all, what is generation, in the higher orders of animals, but an actual inoculation? What is particularly worthy of observation, is the property inherent in every particle of the virus, however minute, of regenerating itself, and reproducing a series of identical phenomena. This series of phenomena is the same, without regard to the condition of the subject inoculated; thus, the vaccine virus has been inoculated in individuals tainted with the highest degree of scrofulous or syphilitic cachexy, and the progress of the pustules has been the same as in healthy individuals. Pus, taken from them, and inoculated in healthy individuals, has given rise to an eruption as regular and normal as possible. Thus, it is demonstrated that virus have a distinct and independent power of reproduction. This is not all; contagious principles, affecting an individual simultaneously, have been seen to produce, for example, both the variolous and vaccine eruption, which progressed together, without influencing, or, in the slightest degree, interfering with each other.

If the intensity of action of contagious principles be not proportionate to the number of individuals affected, the same cannot be said with regard to infectious diseases. Here, the symptoms become more developed, more formidable, in proportion to the number of the sick; and this is understood, by reflecting that we are constantly alimenting and increasing the activity of a focus.

Certain contagious principles pass, by means of inoculation, not only from man to man, but from certain animals to man, as has been lately shown with respect to glanders. Among these principles, are happily some which preserve man from other contagious virus, as the vaccine matter; once communicated to man, it may be indefinitely transmitted to other individuals. But there are some, as rabies, which man has never transmitted to his fellow man, and which appear to perish with him; at least, so far, no authentic fact has proved this communication. And, according to several observers, in the canine race itself, the virus rabiei is not transmissible by a third inoculation. It is scarcely necessary to remark, that certain contagious principles take but once upon the same individual, while the effects of others are repeatable at pleasure.

A question of serious importance has been lately stated, with respect to certain virus. Is it proved that some among them have undergone a kind of degeneracy?—have their properties become enfeebled with time? This has been advanced with regard to the vaccine virus. It was first inferred from an inferior development of pustules and cicatrices. At the present day, more important facts are brought to bear upon the question, connected with the preservative efficacy of the virus. Numerous statistical tables have been cited, and the impression is entertained that subjects, once vaccinated, become again liable to contract variola, after the lapse of a considerable time—say, eight or ten years. The necessity of revaccination is a well established point.

The general effects of contagious principles upon the economy, act chiefly upon the fluid. The blood, in many cases, is evidently altered. In certain diseases of this class, certain symptoms show themselves upon the skin, which lead to the belief in a kind of depuration or elimination of certain morbific agents with regard to other virus; the first phenomena are local, and attack first the parts directly contaminated, as in syphilis; afterwards other organs and tissues, when absorption has had time to take in the noxious principle. Hence the idea of destroying this, before its introduction into the economy. Finally, which is the last term of the action of the virus, every part of the economy may be attacked, when the disease takes the name of constitutional.

